# The Neuroanatomical Bases of Pedophilia and the Importance of Distinguishing Genuine vs. Acquired Types: A Systematic Review

**DOI:** 10.5964/sotrap.6989

**Published:** 2023-05-05

**Authors:** Christian C. Joyal

**Affiliations:** 1 International Center of Comparative Criminology, University of Montreal, Montreal, QC, Canada; 2 Philippe-Pinel National Institute of Legal Psychiatry, Montreal, QC, Canada; 3 Department of Psychology, University of Quebec at Trois-Rivieres, Trois-Rivieres, QC, Canada; Centre for Criminology (Kriminologische Zentralstelle – KrimZ), Wiesbaden, Germany

**Keywords:** acquired pedophilia, brain damage, child sexual abuse, impulsivity

## Abstract

Neurological cases of child sexual abuse (acquired pedophilia) are sometimes used as evidence for the neuroanatomical bases of pedophilia. However, these cases seem to represent a more general syndrome of impulsivity or hypersexuality than a true modification of sexual interests. Therefore, acquired pedophilia may not be adequate to investigate the neurological correlates of pedophilia. The main goal of this study was to systematically review cases of acquired pedophilia to explore the possibility that they are more closely associated with generalized behavioral impulsivity or hyperactivity than a late onset sexual interest toward children. Following the Preferred Reporting Items for SysteMAtic reviews (PRISMA) guidelines, 64 cases of acquired pedophilia were identified. All but one were men. As expected, the mean age of onset for acquired pedophilic behaviors was higher than 50-year-old (M = 52.8-y.-o., SD = 15.6), most cases committed various additional sexual and nonsexual impulsive acts, and only a minority (19%) showed premorbid pedophilic interests. Brain damage mostly involved basal fronto-temporal regions associated with sexual, but also impulsive behaviors. It is concluded that acquired pedophilia should not be used as evidence for the neurological bases of genuine pedophilia. Psychiatric diagnoses of pedophilic disorder would also benefit from adding an exclusion criterion based on neurological etiology. Future investigations are required to determine why acquired pedophilia is almost exclusively observed in men.

“To locate the damage which destroys speech and to locate speech are two different things.”

Hughlings Jackson, 1874

“The frontal lobes exert an inhibiting or constraining influence on what Pavlov called ‘the blind force of the subcortex’—the urges and passions that might overwhelm us if left unchecked.”

Oliver Sacks, 2019

Given their theoretical and clinical implications, neurological correlates of pedophilia are intensively investigated (Jordan et al., 2020; Joyal et al., 2019; Mohnke et al., 2014). A traditional approach to study the neurological bases of behavior is to observe the overt consequences of focal brain damage. Brain-behavior associations based on classical neurological cases allowed important discoveries in the field of neuropsychology, such as the link between the ventro-orbital frontal cortex and behavioral inhibition (the case of Phineas Gage; Harlow, 1848), the contribution of lateral frontal cortical regions to speech (the case of Mr. Leborgne or Tan; Broca, 1861), and the role of bilateral hippocampi in anterograde memory (the case of Henry Molaison or HM; Scoville & Milner, 1957). Although such cases were initially used to localize cognitive functions within the vicinity of the damaged area (e.g., situating articulate language in the third circumvolution of the left frontal lobe; Broca, 1861), these simplistic brain-behavior associations were gradually abandoned with the realization that cognitive functions depend on the integrity of complex cortico-subcortical neural networks. Intriguingly, however, neurological cases of pedophilia are sometimes used to infer the neurological bases of genuine pedophilia (e.g., Alnemari et al., 2016; Perrotta, 2020; Poeppl et al., 2015). As underlined by Scarpazza and colleagues (2021), that outdated approach is commonly used to investigate the neural bases of pedophilia, i.e., the study of patients who developed pedophilic urges and/or behaviors following a brain injury (the so-called acquired pedophilia or sexual behaviors toward children emerging as a consequence of a neurological disorder; Camperio Ciani et al., 2019). The temporal conjunction between brain damage or dysfunction and the emergence of pedophilic behaviors might be interpreted as evidence that the affected brain region is causally related with sexual interests in children. For instance, Mendez and Shapira (2011) suggested that “clarification of the mechanisms and pathophysiology of pedophilic behavior among patients with brain disease could shed light on the nature of [genuine] pedophilia and help develop effective, targeted interventions for this egregious behavior” (p. 1092). Similarly, Miller and colleagues (1986) argued that “[Neurological cases] offer meaningful insights into the anatomy and physiology underlying normal sexual behaviour and provide important evidence regarding the neurological basis of aberrant sexual behaviour” (p. 867). More recently, Lopes and colleagues (2020) stated that “a better knowledge of the injured brain regions that have been related to the emergence of pedophilia may inform upcoming research on the neurobiology of developmental pedophilia” (p. 103).

Some years ago, we argued that most cases of neurologically acquired paraphilia represent signs of a more generalized syndrome of impulsivity or hypersexuality, not true modifications of sexual interests (Joyal et al., 2007). Concerning acquired pedophilia more specifically, Mohnke et al. (2014) also concluded that “in none of the above cited cases, brain pathology specifically led to paedophilia. Rather, in the majority of cases child sexual abuse occurred in the context of hypersexuality, broader changes in personality, impulse control problems and/or neuropsychological deficits” (p. 8). The main goal of the present study was to thoroughly review and update neurological cases of pedophilia to determine to what extent they truly represent acquired pedophilia or, more simply, a sign of acquired impulsivity.

Cases of acquired pedophilia also lead to suggestions that identifying cerebral (damaged) regions associated with pedophilic behaviors should help developing chemical or surgical intervention (Lopes et al., 2020; Mendez & Shapira, 2011), which might (perhaps wrongfully) be applied to genuine pedophilia (e.g., Prado et al., 2021; De Ridder et al., 2009; for extreme examples, see Hitchcock et al., 1972; Roeder, 1966; Schmidt & Schorsch, 1981). Given the importance of establishing the neurological bases of pedophilia (both for theoretical and clinical reasons) and their psychiatric (diagnostic criteria) and legal (criminal responsibility) implications, this study will critically review the available literature concerning acquired pedophilia.

## Brain Damage, Deviant Sexual Behaviors, and Impulsivity

Acquired and genuine (or developmental) pedophilia might be confounded, especially for professionals not working in forensic psychiatry (e.g., psychologists, physicians, lawyers). For instance, Baird (2020) suggested that “there are cases in which there is a clear association between a neurological condition and paedophilia or a paedophilic disorder. Such cases offer critical evidence for the role of specific brain regions in the sexual neural network” (p. 107). That statement may lead the reader to believe that neurological cases of pedophilia could be used to infer the neurobiological bases of pedophilia. However, Baird also stated (correctly) that “those who do develop paedophilia in the context of a neurological condition typically show general behavioural ‘disinhibition’” (p. 113). Indeed, a growing number of authors consider acquired pedophilia as a symptom of a larger disinhibition syndrome (Kruger & Kneer, 2021; Scarpazza et al., 2021). For instance, after reviewing 19 cases of acquired pedophilia, Scarpazza and colleagues (2021) reported that they all suffered from a more general impulse control syndrome. Therefore, the study of acquired pedophilia might contribute minimally to our understanding of the neurobiological bases of genuine pedophilia. From a clinical perspective, persons with acquired pedophilia seem to present a more generalized behavioral disinhibition pattern or a more generalized diminution of sexual target selectivity (hypersexuality). From a legal perspective, these neurological cases seem to have lost their volitional control while keeping the ability to distinguish wrong vs. good behaviors and their consequences (Gilbert & Focquaert, 2015). These impressions should be further confirmed, however.

## The Psychiatric Diagnosis of Pedophilic Disorder

To blur a little more the picture, the mere presence of a repetitive behavior (child sexual abuse), without evidence of sexual preference or interest for children (e.g., corresponding sexual fantasies) is sufficient to receive a diagnosis of pedophilic disorder. Currently, the pedophilic disorder diagnosis is based on two main criteria: A) the presence of recurrent, intense sexual arousing fantasies, sexual urges, *or behaviors* [emphasis added] involving a prepubescent child or children (generally age 13 or less) during a period of six months or more (American Psychiatric Association [APA], 2013) or a sustained, focused, and intense pattern of sexual arousal manifested by persistent sexual thoughts, fantasies, urges, *or behaviors* [emphasis added] involving prepubertal children (World Health Organization [WHO], 2022), and; B) having committed the corresponding *behavior* [emphasis added], being distressed by the arousal (APA, 2013; WHO, 2022), or having interpersonal difficulties because of it (APA, 2013). Given that important criteria such as intensity, sustainment, and focus are not defined or operationalized in these official definitions of pedophilia, the sole presence of child sexual abuse behaviors is sufficient to give a diagnosis of pedophilic disorder in persons with neurological damage, with important implications in forensic context (Gilbert & Focquaert, 2015).

Moreover, contrarily to most diagnoses of the DSM-5 (e.g., depressive disorders, anxiety disorders, obsessive-compulsive disorders, or psychotic disorders), the pedophilic disorder does not include a differential subcategory (“due to another medical condition”) or an exclusion criterion (“the symptoms are not attributable to the physiological effects of a substance or to another medical or neurological condition”) to distinguish neurological etiology. The diagnosis of Intermittent Explosive Disorder, for instance, has the exclusion criterion of neurological origin (e.g., head trauma or Alzheimer’s disease; APA, 2013). For unspecified reasons, this is not the case for paraphilic disorders, including pedophilia.

Although an important distinction is made between child sexual abuse (the behavior) and pedophilia (fantasies, early onset, sexual preference for children) in forensic psychology, sexology and criminology (Seto, 2019), this nuance is commonly overlooked in neurology (Münch et al., 2020). However, a growing number of authors stress the difference between genuine (paraphilic preference, not only behaviors, which usually emerge during adolescence or young adulthood) and acquired pedophilia (e.g., Camperio Ciani et al., 2019; Scarpazza et al., 2021). As expected, classic signs of neurological disorders distinguish men with genuine pedophilia from those with acquired pedophilia (e.g., no criminal premeditation, no history of sexual crimes, being older than 50-y.-o, no attempt to conceal, giving spontaneous confession; Camperio Ciani et al., 2019). In addition, acquired pedophilia has a clear neurological etiology, by definition (contrarily to genuine pedophilia, with unknown neurological bases), and most patients have no history of premorbid pedophilic interest (with few exceptions; Prado et al., 2021; Mendez et al., 2000; Simpson et al., 1999).

It is sometimes suggested that persons with acquired pedophilia already had premorbid sexual interests in children, which were released by the brain damage (disinhibiting a predisposition; Prado et al., 2021; Mendez & Shapira, 2011). These cases would explain why the vast majority of patients with neurological disorders do not abuse children and why the vast majority of persons with acquired pedophilia are men (just like genuine pedophilia). However, these cases seem to be rare, although their prevalence is not clear in the literature. Another goal of this review was to estimate the proportion of persons with acquired pedophilia who had pedophilic interests prior to the brain damage.

## Method

**Figure 1:**
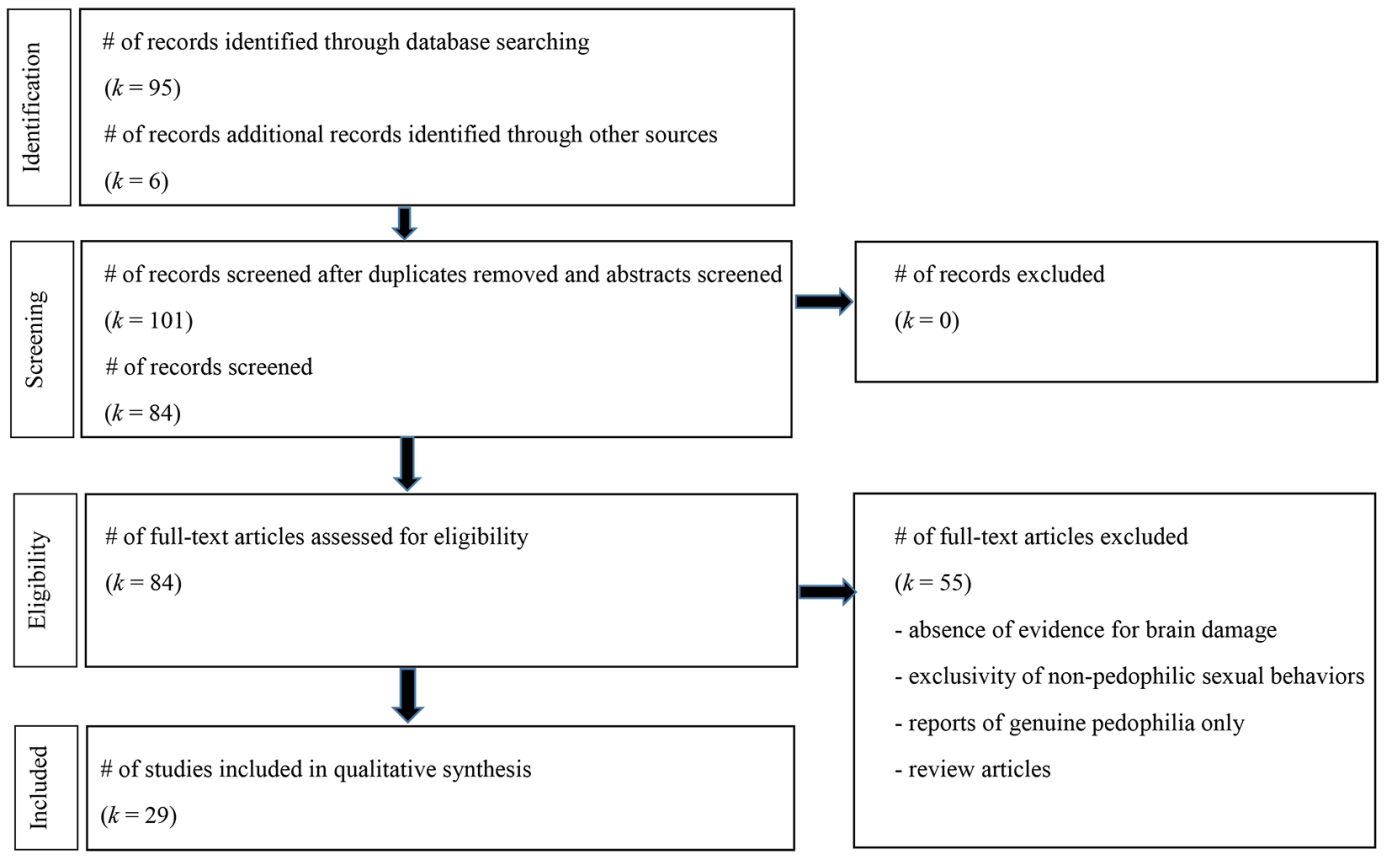
Flow Chart of Article Search Following PRISMA Guidelines

Following PRISMA guidelines (Liberati et al., 2009), a systematic literature search was conducted using Pubmed, PsychInfo, Scopus, ProQuest Dissertations and theses, and Google Scholar databases until June 2021 with the keywords “acquired” or “neurol*” or “brain injury” or “brain damage” and “pedophil*” or “child sex* abuse”. All references included in each article, all references citing each article, and all related papers were consulted. Reports published in English, French and German were reviewed. Exclusion criteria were an absence of evidence for brain damage in the case reported (e.g., cardiovascular conditions without neuroimaging), exclusivity of nonpedophilic sexual behaviors (e.g., fetishism, transvestism), studies based on genuine (developmental) pedophilia only, review papers (no inclusion of original data), and duplicated references (the same study reported in different forms; Figure 1).

## Results

A total of 64 cases of acquired pedophilia reported in 29 publications were identified (see Table 1). Only one case was a woman (Ortego et al., 1993). As expected, the mean age of onset for pedophilic behaviors was relatively elevated (*M* = 52.8-y.-o., *SD* = 15.6) and most cases committed various other sexual and nonsexual impulsive acts. When premorbid sexual interest (*n* = 26) were documented, the majority of cases did not show pedophilic interest behaviors prior to the brain injury (*n* = 21 or 81%), with only five cases having pedophilic interests predating brain damage (premorbid sexuality was not documented in the rest of the cases).

**Table 1:** Case Reports of Neurological Patients With Associated (Following/Resulting) Pedophilic Behaviors

Reports	PP	N (A)	Main official diagnosis / index crime	Brain damage / neurological diagnosis	Other sexual behaviors	Non sexual impulsivity signs
Alnemari et al., 2016	NR	1NS(~22)	Sexual desire toward children and teenagers	Left basal frontal encephalomacia Left temporal encephalomacia	Not mentioned	Attention deficits Irritability Behavioral changes
Berlin & Coyle, 1981	NR	4♂(NR)	Pedophilia	Cortical atrophy (CAT scan)	Not mentioned	Not mentioned
Berger et al., 2003	Yes	1♂(57)	Repeated sexual abuse of a 10-y.o. boy	Parkinson’s disease/dopamine agonist	Fetishism Molesting a 6-y.o. girl	Not mentioned
Blumer, 1970	NR	1♂(41)	Sexual interest for15 y.o. nephew	Right temporal lobectomy	Hypersexuality Increased sexual preoccupations Obscene language	Marked irritability Uninhibited foul talk Candy craving
Burns & Swerdlow, 2003	No	1♂(40)	Pedophilia (child pornography)	Right orbitofrontal (tumor)	Sexual advances toward stepdaughter Prostitution solicitation Sexual advances toward tospital staff and patients	Not mentioned
Casanova et al., 2002	NA	1♂(31)	Sexual perversion	Hippocampal atrophy (pyramidal cells) and schizophrenic disorder (age 69)	Bestiality Sibling incest Frequent masturbation Bisexual behaviors	Compulsive stealing General impulsiveness
(idem)	NA	1♂(57)	Pedophilia	Hippocampal atrophy (pyramidal cells) and bipolar disorder (age 85)	Bestiality Exhibitionism Transvestism Repetitive indecent advances Bisexual behaviors	Bipolar disorder
Devinsky et al., 2010	No	1♂(51)	Began using child pornography	Right posterior temporal resections	Hypersexuality Coprophilia Increased masturbation Compulsive pornography	Hyperphagia Irritability Distractibility
Dimitrov et al., 1999	No	1♂(50)	Rape of a child	Right frontal ventromedial lesion	Big appetite for pornography Stories about sex with minors	Socially disinhibited Unable to handle money Unable to handle jobs Shoplifting
Fairweather, 1947	NR	24♂(NR)	Offences against little girls	Encephalitis lethargica/Post-encephalitic parkinsonism	Varied Indecent exposure Sexual assault	Varied Impulsive outbursts Violent outbursts Impulsive recklessness Self-mutilation
Frohman et al, 2002	No	1♂(36)	Sexual advances to a 12-y.-o. girl Sexually assaulting another minor	Multiple Sclerosis	Asking sexually explicit questions of strangers Masturbating 10-12 times daily Touching breasts of strangers Sexual assault of an adult woman	Behavioral disinhibition Began chain-smoking
Fumagalli et al., 2015	No	1♂(71)	Pedophilia	Traumatic brain injury/lesions Right ventromedial frontal lobe Left frontopolar area Olfactory trigone Right mesial temporal lobe	Exhibitionism Frotteurism Voyeurism	Mild dysexecutive syndrome Behavioral impulsivity Aggressive personality
Gilbert & Vranič, 2015	No	1♂(48)	Physical assault and sexual harassment of pubescent step-daughter	Left frontal lobe (large tumor)	NR	Occasionally mildly aggressive
Kolářský et al., 1967	NR	1♂(NR)	Pedophilia	Temporal lobe epilepsy Perinatal head trauma	NR	NR
Krafft-Ebing, 1886	NA	1♂(20)	Child sexual abuse (4-y.-o. girl)	Multifocal lesions Bilateral frontal lobes First and second temporal convolutions Parts of the occipital convolutions	Bestiality	Violence (killed the victim) Antisocial behaviors
Lesniak et al., 1972	No	1♂(60)	Pedophilia	Right basal frontal lobe (tumor)	Incest Exhibitionism Bestiality Coprophilia Sexual harassment	Irritability Threatening Aggressiveness
Mendez et al., 2000 (also in Mendez & Shapira, 2011)	Yes	1♂(60)	Pedophilia (stalking, accosting, and attempting to molest children)	Focal hypometabolism in the right inferior temporal region (Frontotemporal dementia)	Exhibitionism aiming children Increased sexual activities Increased conjugal demands	Intrudes conversations Intrudes offices Hoarding Hyperphagia Compulsive behaviors Aggressiveness
(idem)	Yes	1♂(67)	Pedophilia (repeated child molestation)	Bilateral hippocampal sclerosis	Increased sexual drive Flirt with female examiners Exhibitionism	NR
Mendez & Shapira, 2011 (in addition of Mendez et al., 2000)	NR	1♂(67)	Sexual advanced toward young daughters	Frontal lobe atrophy (bilateral) Bilateral frontotemporal hypoperfusion (Frontotemporal dementia)	Hypersexual with wife Touch breast of pictured women	Disinhibited behaviors Problems at work Compulsions Lost financial judgment Hyperorality
(idem)	No	1♂(76)	Touched children sexually	Bilateral temporal lobe atrophy (Probable Alzheimer’s disease)	Making sexual comments to children	Disinhibited behaviors Too familiar with strangers Socially inappropriate
(idem)	NR	1♂(82)	Sexually inappropriate with young stepdaughter	Lacunes, left caudate head Lacunes, right globus pallidus Hypometabolism, right cingulate gyrus (Vascular dementia)	Spend hours masturbating Touch people inappropriately	Disinhibited behaviors Poor impulse control Attention deficits Increased appetite
(idem)	No	1♂(59)	Child pornography	Parkinson’s disease/dopamine agonist (No MRI anomaly)	Constantly seeking sex Patronize massage parlors Recurrent use of prostitution	None
(idem)	NR	1♂(32)	Sexually touched a 6-y.-o. girl	Huntington’s disease	NR	Behavioral impulsivity Aggressiveness Attention deficits
(idem)	No	1♂(59)	Inappropriately touched 5-y.-o granddaughter	Right pallidotomy (Parkinson’s disease/dopamine agonist)	Sexually forced his wife Compulsive demands for oral sex Use of prostitution Excessive use of pornography Sexual advances to wife’s friends Masturbation viewing a picture of granddaughter Intrusive sexual thoughts	NR, although excellent attention and executive functions are demonstrated. Impulsivity seems to be limited to sexual behaviors (hypersexuality).
Miller et al., 1986	No	1♂(50)	Propositioning children in the neighborhood	Infiltrating hypothalamic glioma	Sexual advances to 7 y.o. daughter Sexual advances to daughter’s friends Frequent, increasing public sexual advances towards young children Begins collecting pornography Discusses sex almost continuously Frequently embarrass his wife	Lost financial judgment Bankruptcy
Miller & Cummings, 1991	No	1♂(64)	Had sex with a 16-y.o boy	Right frontal arteriovenous malformation (including septum, basal ganglia, hypothalamus)	NR	NR
Müller et al., 2003	No	1♂(28)	Pedophilia (repeated child sexual abuse)	Right prefrontal hypometabolism (motorcycle accident/head trauma)	NR	Impulsive Behavior (including pre-morbid) Violence/impulsiveness Antisocial personality Intermittent explosive disorder Aggressive outbursts
Ortego et al., 1993	No	1♀(39)	Sex with a minor female	Multiple sclerosis (basal-orbital frontal cortex, septum, thalamus, hypothalamus, temporal cortex, brainstem, cerebellum)	Offering sex to 15 y.o. boy Hypersexuality Bestiality Voyeurism Exhibitionism	NR
Poeppl et al., 2015	Yes	1♂(45)	Obsessional use of child pornography	Bilateral frontotemporal theta dysrhythmia (Epileptiform brain activity)	NR	Psychopathic traits
Prado et al., 2021	Yes	1♂(69)	Attempts to kiss a 7-y.o girl and granddaughter	Global cerebral atrophy Dorsomedial left frontal lobe Anterior left temporal lobe Probable behavioral frontotemporal dementia	Attempts to kiss 6 and 9-y.o. granddaughters Attempted seduction, 7-y.o. boy Premorbid incest with daughter	Several antisocial behaviors
Rainero et al., 2011	No	1♂(49)	Sexual arousal and urges toward 9 y.o. daughter	Bilateral frontal lobe atrophy Frontal bilateral hypoperfusion (Frontotemporal dementia)	NR	Verbal and physical violence
Regestein & Reich, 1978	No	1♂(56)	Repeated sexual activity with 8-y.-o. nephew	Right frontal craniotomy (Tumor)	Inappropriate with a 5-y.-o. girl Sexual solicitation of a 14-y.-o. boy Hypersexuality	None (apathy) (depression)
Sartori et al., 2016	No	1♂(64)	Sexually inappropriate behavior towards a female child in a kindergarten	Orbitofrontal cortex compression Optic chiasm compression Hypothalamus compression (Clivus Chordoma)	Pedophilic urges	Impaired at Hayling test Behavioral disinhibition Obsessions-compulsions Stealing in a museum shop
Scarpazza et al., 2018	No	1♂(~70)	Pedophilia (two incidents of sexual behaviors with minor boys one year apart)	Bilateral frontal lobe atrophy (Frontotemporal dementia)	Hypersexuality	Attentional deficits Behavioral impulsivity Lack of consideration for consequences Verbal violence Hyperphagia Kleptomania
(idem)	No	1♂(~60)	Sexual behavior toward a male child	Extended brain damage (right fronto-parietal meningioma)	Repeated masturbation in front of a school	Impulse dyscontrol Impaired sustained attention Impaired inhibition Impaired planning abilities
Simpson et al., 1999	No	2♂(NR)	Child molestation	NR (Traumatic brain injury)	NR	NR
Solla et al., 2006	No	1♂(62)	Sexual interest toward granddaughter	Parkinson’s disease/dopamine agonist	Bestiality (family female dog)	NR

*Note*. Presented in alphabetical order of first authors. N = number of participants; A = age; NR = not reported; NA = not applicable (postmortem neuropathology); PP = premorbid predisposition.

## Discussion

Given the theoretical, clinical and legal importance of studying the neurological substrates of pedophilia, case studies of acquired pedophilia are commonly used as neuroanatomical models. However, the results of this review suggest that acquired pedophilia is more closely related with behavioral impulsivity in general than sexual deviance in particular. These results have several implications, which will be described in more detail below.

### Brain Damage: Sexual Deviance, Hypersexuality, or General Disinhibition?

Considered alone, pedophilic behaviors following a brain injury could be interpreted as the etiology of a new sexual interest in children. When the complete clinical picture is considered, however, these pedophilic behaviors are usually one of many symptoms of a general disinhibition syndrome, or at least of a hypersexuality-related disorder. As stressed by Starkstein and Robinson (1997) fifteen years ago, “several studies in patients with closed head injuries, brain tumors, stroke lesions, and focal epilepsy have demonstrated a significant association between disinhibition syndromes and dysfunction of orbitofrontal and basotemporal cortices” (p. 108). The results of the present review suggest that a similar phenomenon applies to most cases of acquired pedophilia.

Three main neurobiological theories currently attempt to explain pedophilic offending (not to be confounded with nonoffending pedophilia): the frontal-dysexecutive model, the temporal-limbic model, and the dual-dysfunctional theory (Dillien et al., 2020). According to the frontal-dysexecutive model, pedophilic behaviors result from frontal lobe anomalies, leading to disinhibited behaviors. According to the temporal-limbic model, damage to the temporal lobe lead to atypical sexual interests or hypersexuality, given the limbic system involvement in emotion and motivation regulation, including sexual behaviors. The dual-dysfunctional theory merges the two models, assuming a dysfunction in both the frontal and the temporal lobes. As stressed by a growing number of authors, however, these theories explain hypersexuality and disinhibition, but not pedophilic interests *per se* (Caffo et al., 2021; Jordan et al., 2020; Kruger & Kneer, 2021). In view of the present results, the same conclusion can be drawn about acquired pedophilia. Interestingly, a recent brain imaging meta-analysis based on 436 men with a pedophilic disorder failed to find any structural difference compared with 449 controls (Scarpazza et al., 2021). Therefore, anomalies of fronto-temporal regions appear to be more closely associated with child sexual abuse (acting out) than pedophilia (Dillien et al., 2020). Given that several cases studies reviewed here omitted to record (or report) nonsexual signs of impulsivity, these aspects should be considered in future investigations of acquired pedophilia.

We previously reviewed and updated the neurophenomenological model of sexual arousal (Stoléru et al., 1999) through a meta-analysis of neuroimaging data (Stoléru et al., 2012). This model integrates five neuro-bio-psychological components of sexual arousal: 1) cognition (i.e., attention, appraisal, imagery); 2) emotion; 3) motivation; 4) physiology (autonomic and endocrine responses), and; 5) inhibition. Although the cognitive component is the most complex, it depends crucially upon the inhibitory component (modulatory function), which therefore plays a prominent role in the frequency and target of sexual arousal. The inhibitory component includes three subcomponents: 1) latency inhibition (occurring during periods between sexual arousal and blocking its emergence); 2) devaluation (cognitive inhibition decreasing the sexual relevance of a stimulus when the context or the target is inadequate); and; 3) withholding an action (behavioral inhibition of the overt expression of sexual arousal). It is worth noting that neurological substrates of these inhibitory subcomponents are commonly affected in cases of acquired pedophilia (as described in Table 1; see also Kruger & Kneer, 2021; Mohnke et al., 2014), including the lateral orbitofrontal and temporal cortices (latency inhibition), the medial orbitofrontal cortex (devaluation), and the basal ganglia (caudate nucleus; withholding). These results are in line with the suggestion that a deficit in one or more inhibitory components of sexual arousal explains a significant part of acquired pedophilia. Although this type of pedophilia may meet the DSM-5 criteria, it is usually not associated with the essential features of a paraphilia (i.e., sexual preference and sexual fantasies). This conclusion argues for the addition of neurological damage as an exclusion criterion for the pedophilic disorder, just as it is the case for most other DSM-5 diagnoses.

As stressed more than 10 years ago by First and Halon (2008), “diagnostically, one error that can result in a false positive diagnosis (i.e., opining that a paraphilia is present in the respondent when it is not) is to base the diagnosis solely on the presence of the criminal sexual behavior without evidence causally connecting that behavior to the paraphilic arousal pattern” (p. 446). Similarly, the Working Group on the Classification of Sexual Disorders and Sexual Health of the ICD-11 rightfully stressed that a history of sexual behavior involving children *should not be sufficient* [emphasis added] to establish the diagnosis of pedophilic disorder because an essential feature of the condition is a sustained, focused and intense pattern of sexual arousal to prepubescent children (Krueger et al., 2017). Krueger et al. (2017) specifically stated that when only behaviors are present, other causes should be considered because of the possibility of nonsexual or other explanations for these behaviors (p. 1537). It could be argued that a brain injury should be considered as an exclusion criterion (i.e., other explanation) for the pedophilic disorder. However, given the lack of operationalization and instruments for assessing the criteria of sustained, focused and intense interests, many cases of pedophilia are still based solely on behaviors (especially in neurology), without the required sexual arousal pattern, preference or specificity.

It would be interesting to assess sexual fantasies of men with acquired pedophilia. Although seldom reported in existing studies, there is a possibility that acts of child sexual abuse following brain injury are not only unpremeditated, but also unassociated with corresponding sexual fantasies. The presence of these sexual urges without corresponding sexual fantasies might further help distinguishing acquired pedophilia from genuine pedophilia. Developing and providing validated measures of such important criteria for a pedophilic disorder as intensity and focus of sexual interests would also significantly contribute to the confirmation of the diagnosis in neurological cases of child sexual abuse.

### Legal Implications

The study of acquired pedophilia has important legal implications because the condition is usually associated with a special neuropsychological pairing, i.e., a lost of volitional control with preserved insight, awareness and/or moral. The preservation of insight, in turn, commonly lead the justice system to consider persons with acquired pedophilia to be responsible of their acts, with all the legal consequences associated with these decisions (Gilbert & Focquaert, 2015). Although offenders with acquired pedophilia might indeed be well aware of the wrongfulness of their acts (mens rea, “guilty mind”), the brain damage explains in large part their acting out because it results from a significant reduction of volitional control and behavioral inhibition (actus reus, “guilty act”). Therefore, the difference in etiology, motivation, and context between acquired and genuine pedophilia should have important legal implications (Scarpazza et al., 2018). As underlined by Gilbert and colleagues (2016), an impaired capacity to control one’s behavior and urges due to neurological damage should mitigate criminal responsibility. It could also help choosing more appropriate treatment plans (e.g., more closely related with neurological disorders than paraphilic disorders).

### Acquired Pedophilia: A Male Disorder

Although sexual interest in children is relatively rare in women, it is reported by a small minority among the general population (Wurtele et al., 2014). One notable result from this review is the quasi exclusivity of male cases. If damage to fronto-temporal neural networks simply provoke a generalized disinhibition (as suggested here), including sexual urges toward children, both men and women with similar brain damage should show acquired pedophilia. This sex imbalance is reminiscent of that observed in most cases of paraphilic disorders. It remains possible that neurobiological factors (e.g., neurohormones, genes) closely associated with sex drive and male gender (e.g., testosterone, X chromosome) play a mediating role between brain damage and sexual abuse of children leading to acquired pedophilia. It remains also possible that damage to subcortical structures associated with sexual behaviors (e.g., amygdala, hypothalamus) distinguishes disinhibition syndromes with vs. without pedophilic manifestations. Future interdisciplinary investigations will help disentangling these etiological factors of acquired pedophilia.

### Conclusion

Studies in behavioral neurology generally led to the conclusion that acquired pedophilia has three main types of origins, associated with lesions to three main cerebral regions: 1) a general disinhibition syndrome induced by basal-orbital frontal damage; 2) hypersexuality following temporal subcortical damage (e.g., hypothalamus, hippocampus) and; 3) a true modification of sexual interest, associated with temporal amygdala lesions (Mendez & Shapira, 2011). In view of the present results, it is suggested that disinhibition and hypersexuality but not true modification of sexual interest are associated with acquire pedophilia.

As recently concluded by Kruger and Kneer (2021), “for a long time, a so-called ‘symptomatic’ sexual deviance (including pedophilic behaviors) was reported after brain injury […]. These observations may not necessarily reflect a genuine change of sexual preference or a disclosure of a latent paraphilic disorder, but may indicate the importance of intact brain function for controlling sexual behaviors, particularly temporal and frontal regions. Sexual disinhibition might explain a non-specific broadening of sexual behaviors (like a disturbance of a sexual filter system) that may include deviant ones.” (p. 72). The results of this review concord with that conclusion. It could also be suggested that damage to basal fronto-temporal regions induce a disturbance of a general filter, prompting both sexual and nonsexual impulsive acts, including pedophilic behaviors. Therefore, cases of acquired pedophilia may not be adequate (or at least optimal) to infer the neurological bases of pedophilia.
